# Who to Be Treated: Nomogram Using Self-Reported Periodontal Screening Instrument among English-Speaking Adults in Multi-Ethnic Singapore

**DOI:** 10.3390/jpm12060931

**Published:** 2022-06-04

**Authors:** Christina P. C. Sim, Huihua Li, Marco A. Peres

**Affiliations:** 1 Department of Restorative Dentistry, National Dental Centre Singapore, 5 Second Hospital Avenue, Singapore 168938, Singapore; 2 Oral Health Academic Clinical Programme, Duke-NUS Medical School, National University of Singapore, 8 College Road, Singapore 169857, Singapore; 3 ACP Research Office, National Dental Centre Singapore, 5 Second Hospital Avenue, Singapore 168938, Singapore; li.hui.hua@ndcs.com.sg; 4 Centre for Quantitative Medicine, Duke-NUS Medical School, National University of Singapore, 8 College Road, Singapore 169857, Singapore; 5 National Dental Centre Singapore, National Dental Research Institute Singapore, 5 Second Hospital Avenue, Singapore 168938, Singapore; marco.a.d.a.peres@ndcs.com.sg; 6 Oral Health Academic Clinical Programme, Health Services and Systems Research Institute Singapore, Duke-NUS Medical School, National University of Singapore, 8 College Road, Singapore 169857, Singapore

**Keywords:** severe periodontitis, periodontal disease, disease risk, nomogram, decision curve analysis, self-reported questionnaire

## Abstract

Periodontal disease is a major public health problem. This study aimed to develop a nomogram using a self-reported periodontitis screening instrument in predicting severe periodontitis (SP), defined by the World Workshop on Classification of Periodontal and Peri-Implant Diseases and Conditions, and evaluate its utility in clinical setting. An Akaike information criterion selected multivariable model was developed to predict SP using a self-reported questionnaire, with a nomogram developed based on its regression coefficients. Discriminatory capability was evaluated by Receiver-operating characteristic curve. Ability to predict SP of individual patients was evaluated with bootstrapping. Decision curve analysis (DCA) was performed to evaluate its potential clinical utility by evaluating clinical net benefit at different thresholds. 58.1% of 155 participants were classified with SP. Older males without tertiary education, with ‘loose teeth’, ‘bone loss’ and ‘mouth rinse use’ had higher SP risk. The nomogram showed excellent discriminatory capability with Area under Curve of 0.83 (95% CI = (0.76, 0.89)), good calibration (intercept = 0.026) and slight overestimation of high risk and underestimation of low risk (slope = 0.834). DCA showed consistent clinical net benefit across the range of thresholds relative to assumption of ‘no patient’ or ‘all patient’ with SP. Our nomogram using a self-reported periodontitis instrument is useful in SP screening in English-speaking Singaporean adults.

## 1. Introduction

Periodontal disease is a chronic inflammatory oral disease and is the 11th most prevalent disease globally [1]. It is characterized by the destruction of the tooth-supporting tissues manifested by loss of clinical attachment and radiographic bone loss [2]. Its development and progression depend on a myriad of local and systemic risk factors [3]. It is also significantly associated with several chronic non-communicable diseases such as cardiovascular diseases, obstructive pulmonary disease, Type 2 diabetes mellitus, rheumatoid arthritis and chronic kidney disease [4,5,6,7]. Severe periodontitis (SP) is the leading cause of tooth loss and has a negative impact on the affected individual’s physical and psycho-social functions [3]. Early detection of severe periodontal disease is important to prevent the development and progress of significant tissue destruction. By 2017, the age-standardized prevalence of SP globally was 9.8%, a 5.8% increase from the year 1990 [8]. For the same period, the corresponding percentage change in the Singapore population was higher at 11.5% [8]. As world population grows due to increased life expectancy and tooth loss prevalence decreases, SP will continue to be a major public health burden [9]. It is thus important to monitor periodontal disease’s development and progression at different levels of the population over time [10].

Self-reported health status has been considered to be an accepted and efficient method for disease prevalence assessment [10]. Results from self-reported surveys indicated however, that socio-demographic parameters and past disease experience influenced individuals’ health knowledge, affecting agreement between clinical examination and self-reported data [11,12].

Validity studies for self-reporting of periodontal disease have been shown to produce inconsistent results [10] as they depend on several factors: participant age, periodontal case definition applied, disease severity, cross-cultural adaptation of questionnaire items, dental services access and utilization [13,14,15]. In 2003, the Centre for Disease Control and Prevention (CDC) and the American Academy of Periodontology (AAP) initiated the CDC Periodontal Disease Surveillance Project which tested eight question-constructs to predict population prevalence for periodontitis [16]. Six of the eight questions were pre-pilot field-tested during the 2004–2006 Australian National Survey of Adult Oral Health (NSAOH), with promising results [17]. Of the six questions, two questions on the use of ‘mouth rinses in the last seven days’ and ‘interdental aids in the last seven days’ had the same meaning but were phrased slightly differently in the NSAOH [17]. Subsequent testing and validation of the set of eight questions was carried out during the United States National Health and Nutrition Examination Survey (NHANES) 2009–2010 cycle and it was shown to adequately estimate the prevalence of periodontitis [15]. Accuracy was further improved with the addition of demographic and risk factor variables, coupled with six sites per tooth full-mouth periodontal examination [18,19]. Higher validity can thus be achieved with a combination of self-reported questions, demographic and risk factor variables [10]. Since then, the validity of self-reporting for periodontitis has been tested in different Western populations [14,15,20,21,22,23], including high-risk populations in rural areas [24], African Americans [25] and post-partum women [26]. Very few studies have been carried out in Asian populations, mainly in China [27] and Japan [28]. In addition, validation studies based on the new periodontitis classification (World Workshop on the Classification of Periodontal and Peri-implant Diseases and Conditions (WWC)) [29] are sparse [23,24].

Nomograms from multivariable logistic models or Cox proportional-hazards regression are a popular visual plot to display the predicted probabilities of an event for decision support [30]. They depict a statistical predictive/prognostic model that generates a probability of a clinical event for a given individual. Nomograms are widely used for cancer prognosis, primarily because of their ability to reduce statistical predictive/prognostic models into a single numerical estimate of the probability of an event, such as death or recurrence, that is tailored to the profile of an individual patient [31]. The current revival in the use of nomograms as diagnostic and prognostic tools attest to their effectiveness in facilitating communication between the healthcare provider and patient [32]. However, their use is still scarce in dental research and dental practice.

To our knowledge, there are no studies evaluating the predictive performance of self-reported oral questions in a multi-cultural South-east Asian population such as Singapore using the nomogram. Singapore is a multi-ethnic multi-lingual society made up of three major ethnic groups (Chinese, Malays and Indians) where language policies operate based on English as the country’s lingua franca. This study aims to test the predictive performance of a nomogram developed from a self-reported periodontal screening instrument in identifying English-speaking Singaporean adults with high risk of SP.

## 2. Materials and Methods

### 2.1. The Setting, Study Population and Sample Size

Study participants were recruited from the appointment list of adults referred for their first consultation visit to either the endodontic, periodontic or prosthodontic units at the National Dental Centre Singapore (NDCS). They received a letter of invitation, followed by a telephone call two weeks later. Eligibility criteria included adults aged ≥ 21 years, have ≥6 teeth, comfortable with speaking English and provided informed consent to participate in the study. Exclusion criteria were non-English speaking adults or those with <6 teeth.

Based on the data reported in the 2003 National Adult Oral Health Survey [33], 60.8% of participants had a CPI score of ≥3. Assuming 60% prevalence of SP, 142 participants were needed to detect an AUC of 0.86 with 95% CI of 0.80 to 0.92. Considering a 10% dropout rate, 158 participants should be recruited (of which 95 would have SP and 63 would be without SP). In a multivariable logistic regression, at least 10 events are required for each coefficient [34]. Our data would allow us to develop a multivariable model which include up to six coefficients given the effective sample size of 63.

### 2.2. Study Design

This cross-sectional study consists of a self-reported telephone interview survey and a clinical oral examination.

### 2.3. Telephone Interview Survey

Trained study coordinators handled the telephone calls to the invited new patients. If the patient agreed to participate, the patient would complete a questionnaire consisting of three domains: socio-demographic, risk factors and periodontal health questions (Table 1). Of the 10 self-reported periodontal health questions, six (gum disease, gum health, gum treatment, loose teeth, lost bone around teeth, tooth doesn’t look right) were those proposed by the CDC-AAP [16] and two (mouth rinse use and interdental aids usage in past seven days) were used in the Australian NSAOH [17]. The remaining two questions were ‘upper tooth count’ and ‘lower tooth count’. Variables such as gender (male/female), age, ethnicity (Chinese/Malay/Indian/Others), education (non-tertiary/tertiary), monthly household income (<SGD $2000/SGD $2000–9999/above SGD $10,000), housing type (public/private housing), smoking history (never/past/current smoker) and diabetes (self-reported diagnosis by a health professional: yes/no) were recorded to explore their influence on SP. Tertiary education referred to completing at least a university education. The questionnaire was hosted in a commercially available online survey platform, Qualtrics^®^ (Provo, UT, USA). The data entry program included logic and range checks. Password protected tablets were used for data collection.

### 2.4. Clinical Examination

The clinical examination consisted of a full mouth periodontal and radiographic examination and medical history. The clinical examiner was blind to the participant’s answers to the telephone interview questionnaire. Clinicians from the three clinical units assigned to examine new patients were calibrated against a senior clinician and provided with oral and written instructions on study details and measurement techniques. To evaluate inter-examiner reproducibility, five subjects not involved in the study, but who met the inclusion criteria, were assessed. The intra-class correlation coefficient for probing depth (PD), a measure of inter-examiner reliability, was 0.91. Periodontal examination was carried out using a periodontal probe (PCPUNC15, Hu Friedy, Chicago, IL, USA), recording PD, gingival recession (GR), plaque index, mobility, furcation involvement and bleeding on probing at six sites (mesio-buccal, mid-buccal, disto-buccal, disto-palatal/lingual, mid palatal/lingual, mesio-palatal/lingual) of all teeth, except for third molars. Measurements for PD and GR were recorded in millimeters, rounded to the nearest millimeter. Clinical attachment levels (CAL) were calculated using the examiner’s measurements of PD and GR. Periodontal disease classification was staged according to the WWC classification [35] where severity was classified into four stages (Stage I, II, III, IV). For clarity, SP refers to Stage III/IV in the WWC classification.

### 2.5. Statistical Analysis

Participants’ characteristics were summarized for each age group. Frequencies and proportions were reported for categorical variables; means and standard deviations (SD) were reported for continuous variables. Univariable logistic regression was carried out to evaluate the effect of self-reported questions on SP. An Akaike information criterion (AIC) selected multivariable logistic regression model was developed to predict SP. Its performance was investigated to evaluate the discriminatory capability to diagnose SP by Receiver-operating characteristic (ROC) curve analysis, with an Area Under the Curve (AUC) reported for the model. AUC value of 0.5 suggests no discrimination to diagnose patients with the disease from the rest; AUC values between 0.5 and 0.7 suggest poor discrimination; AUC values between 0.7 and 0.8 suggest acceptable discrimination; AUC values between 0.8 and 0.9 suggest excellent discrimination and AUC value > 0.9 suggests outstanding discrimination [36].

A nomogram was generated based on the AIC selected multivariable logistic regression coefficients. The Hosmer-Lemeshow goodness-of-fit test was carried out to evaluate the agreement between predicted and overview probabilities. Calibration plots were generated to evaluate the ability of the nomogram to predict SP of individual patients by calculating an optimism-corrected estimate of performance with bootstrapping of 2000 bootstrap set of resamples. The estimated regression slope and intercept dictate the direction of mis-calibration and overall mis-calibration, respectively. Slope of 1 and intercept of 0 denotes perfect calibration, Slope > 1 denotes underestimation of high risk and overestimation of low risk, while slope < 1 denotes overestimation of high risk and underestimation of low risk. Intercept > 0 indicates an average underestimation, while <0 indicates an average overestimation [37].

Decision curve analysis (DCA) was performed to evaluate the potential clinical utility of nomogram application by evaluating the clinical net benefit at different thresholds, which was derived by examining the theoretical relation between the threshold probability of developing an event and the relative value of false-positive and false-negative results as described by Vickers et al. [38]. All analyses were performed using R4.1.1 [39] at two-sided significance level of 0.05.

### 2.6. Ethical Issues

The study protocol, approved by the Singhealth Centralized Institutional Review Board (Ref:2019/2418), was conducted following the Helsinki Declaration, as revised in 2013. All participants gave written informed consent.

## 3. Results

Of the 158 new patients recruited into the study, 155 completed both the telephone interview survey and the clinical examination (77.4% Chinese, 13.2% Indian, 3.2% Malay and 3.2% other ethnicities). 53.5% were males and the mean age of the total sample was 55.8 ± 11.8 years. As 48 (31%) participants were unable to state their monthly housing income, with a higher proportion of private home residents (44%) unwilling to state their monthly housing income compared to public housing residents (25%), the variable ‘monthly housing income’ was not included in any analysis. Patient characteristics by age groups are listed in Table 2.

**Figure 1:**
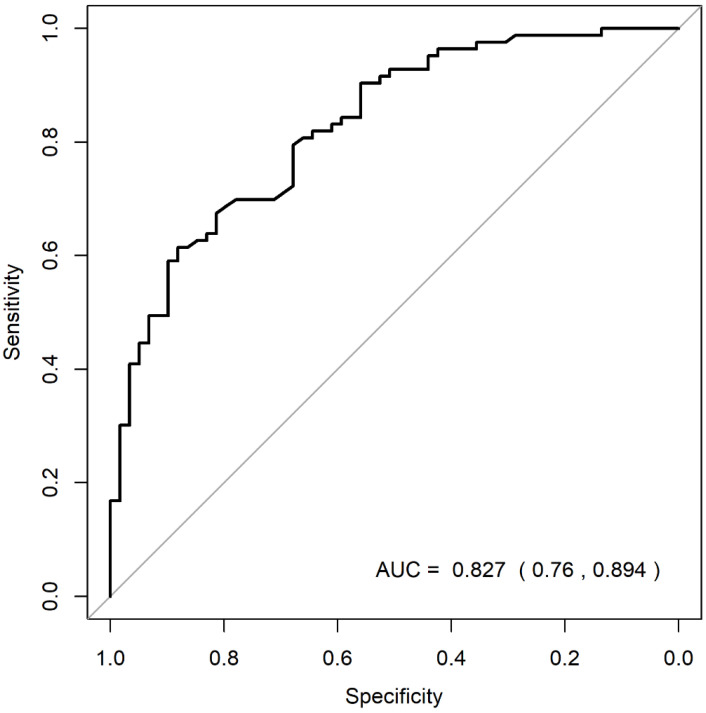
Receiver-operating characteristic curve in predicting severe periodontitis according to WWC classification (Sensitivity = 70.0%, Specificity = 78.0%, Negative Predictive Value = 64.8%, Positive Predictive Value = 81.7%, cut-off (risk) = 59%, nomogram score = 44.3. WWC: World Workshop on the Classification of Periodontal and Peri-Implant Diseases and Conditions).

Questions on age, gender, diabetes status, ‘gum disease’. ‘lost bone’, ‘loose teeth’, ‘tooth doesn’t look right’, ‘gum health’ and ‘mouth rinse use’ individually showed significant effect on SP by univariable logistic regression (Table 3). Based on multivariable logistic regression and using the WWC classification, older non-tertiary educated male participants who reported loose teeth with bone loss around their teeth and used mouth rinses had higher risk of developing SP (Table 3). From the ROC curve analysis, the optimal cut-off risk or predicted probabilities selected was 0.59. This value showed the best balance of sensitivity (70%) and specificity (78%), corresponding to a ‘Total Score’ of 44.3 in the nomogram. At 0.59 cut-off risk, ROC curve analysis showed excellent discrimination in differentiating SP from the rest with an AUC of 0.83 (95% CI = (0.76, 0.89)) (Figure 1).

**Figure 2:**
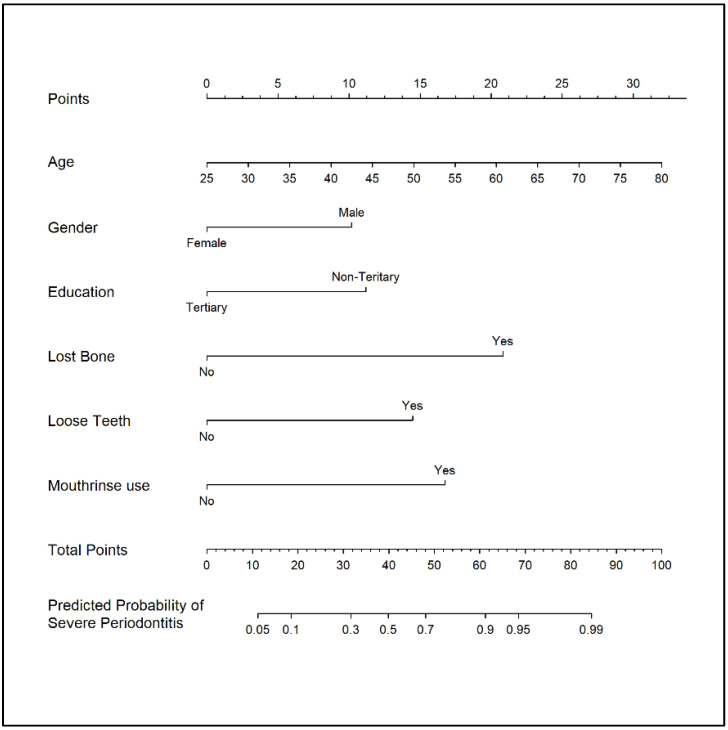
Nomogram to predict severe periodontitis according to WWC classification. To use the nomogram, an individual participant’s value is located on each variable axis, and a line is drawn upward to determine the number of points received for each variable value. The sum of these numbers is located on the Total Points axis to determine the risk of severe periodontitis. (WWC: World Workshop on the Classification of Periodontal and Peri-Implant Diseases and Conditions).

A nomogram (Figure 2) to predict SP was developed based on this model. The nomogram is a predictive tool to evaluate SP risk based on the additive evaluation of individual risk factors of SP. The upper portion of the nomogram (‘Points’) is used to compute the weight of every factor in this nomogram (age, gender, education, ‘loose teeth’, ‘lost bone’ and ‘mouth rinse use’). The sum of these weights is calculated to provide patient-level risk and is reflected on the ‘Total Points’ axis located at the lower portion of the nomogram. For example, the nomogram can be used to predict the risk of developing SP for a tertiary-educated 50-year-old female who has loose teeth and bone loss around teeth but do not use any mouth rinses. Firstly, the score of each factor is computed: the corresponding scores for ‘50 years of age’, female, tertiary education, ‘loose teeth’, ‘bone loss’ and no ‘mouth rinse use’ are 14.5, 0, 0, 14, 21 and 0, respectively. The summation of scores obtained for every factor gives a ‘Total Score’ of 49.5, which is applied to the lower component of the nomogram to determine the individual’s SP risk. If the risk from ‘Total Score’ falls below the cut-off risk of 0.59, the individual will be considered to have low risk of developing SP. Conversely, if the risk falls above the cut-off risk of 0.59, the individual will be classified as a high SP risk. In the given example, the individual has a ‘Total Score’ of 49.5, corresponding to a risk of 0.73 (>cut-off risk 0.59), suggesting the need for a referral for further clinical assessment.

**Figure 3:**
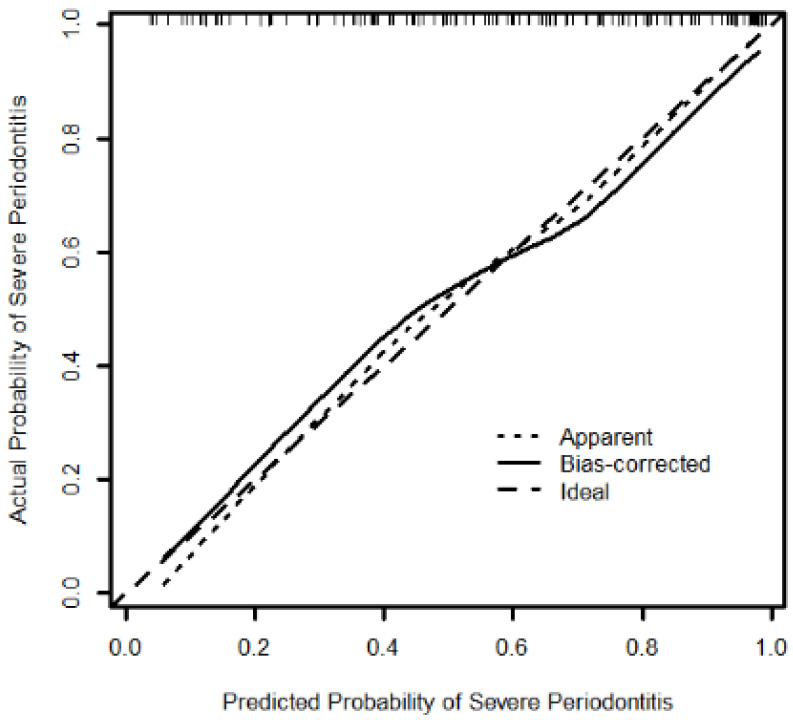
Calibration curves for AIC Selected Model (intercept = 0.026, slope = 0.834). The diagonal dotted line represents a perfect prediction by an ideal model. The solid line represents the performance of the nomogram, for which a closer fit to the diagonal dotted line represents a better prediction. (AIC: Aikaike information criterion).

**Figure 4:**
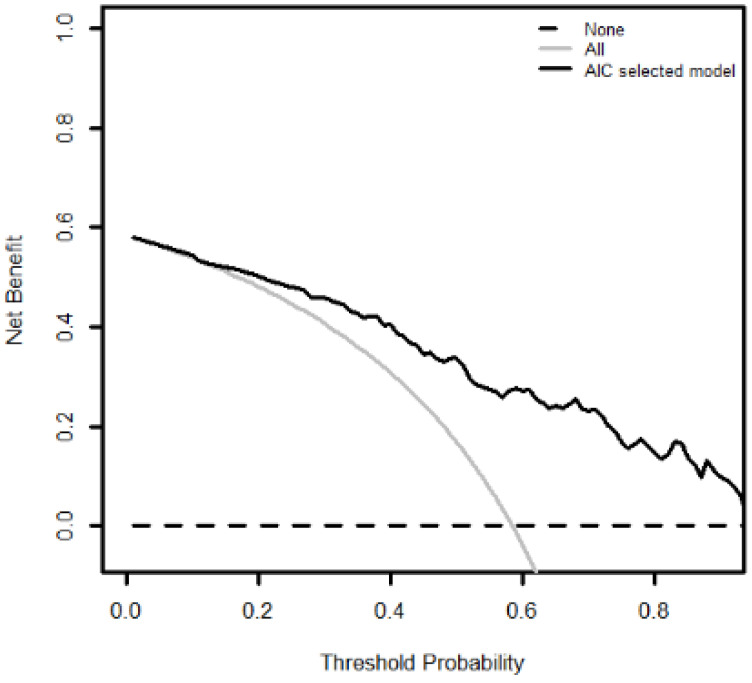
The decision curve plotting of net benefit against threshold probability for nomogram predicting severe periodontitis according to WWC classification. (WWC: World Workshop on the Classification of Periodontal and Peri-Implant Diseases and Conditions).

Logistic regression analysis showed that SP risk increased with increased nomogram score (OR = 1.10, 95% CI = (1.06, 1.14)). The Hosmer–Lemeshow goodness-of-fit tests showed good fit between observed and predicted events using this nomogram (*p* = 0.714). Calibration curve demonstrated good bootstrap estimates of calibration accuracy of nomogram with an intercept close to 0 (0.026) and slight overestimation of high risk and underestimation of low risk (slope = 0.834) (Figure 3). These findings were confirmed by internal validation using bootstrapping (average bootstrap AUC = 0.80). Compared to the assumption of ‘no patient’ or ‘all patient’ with SP, DCA (Figure 4) showed consistent clinical net benefit across a wide range of threshold probabilities.

## 4. Discussion

This study is the first to assess the utility of a self-reported periodontal screening instrument to screen for SP in Singaporean adults and to determine the predictive ability of the nomogram developed from this screening instrument. Our results showed the feasibility of the instrument for SP screening using the WWC classification. Prevalence of clinically diagnosed SP differed across the different age groups. The ≥65 years old age group had the highest proportion of individuals with SP, despite having the least number of teeth.

The self-reported periodontal screening instrument consisted of 10 periodontal screening questions and eight questions relating to socio-demographic and risk factors. Eight of the 10 periodontal screening questions had been previously validated in a representative sample of US adults [15] and in the Australian 2004 NSAOH [17]. To validate the questionnaire in a multi-ethnic society such as Singapore, we tested it on a sample of patients who attended NDCS for their first dental visit and were comfortable with speaking English. Two additional questions on upper and lower jaw tooth counts were added to the present survey to determine the usefulness of self-reported tooth count in SP prediction, as tooth count had shown good predictive ability for SP [40,41]. However, our results showed no significant association of tooth count with SP. Individually, all questions except for housing type, upper tooth count, lower tooth count and interdental aids use, were associated with SP, indicating that the periodontal screening questions used in the National Oral Health surveys in USA and Australia might be useful in predicting SP in a sample of English-speaking multi-ethnic Singaporean adults, as seen in our study population.

Questions on ‘gum disease’ and ‘gum health’ were highly associated with SP, with a significantly higher risk for those with gum disease (OR = 3.26) and lower risk for those who rated their gum health as ‘good/very good/excellent’ (OR = 0.40). This indicated a high self-awareness of one’s own periodontal health, and supported the use of a self-reported periodontal screening instrument as a possible surveillance tool in the local population. Our results concurred with findings from published systematic reviews reporting acceptable validity of the question on self-awareness of gum disease in identifying SP [10,42].

The nomogram developed from the self-reported periodontal screening instrument included three periodontal screening questions (‘lost bone’, ‘loose teeth’ and ‘mouth-rinse use’) and three socio-demographic factors (age, gender and education). It showed good discriminative capability to detect SP with 70% sensitivity and 78% specificity at the cut-off risk of 0.59. Among the three periodontal screening questions, the question ‘lost bone’ had the biggest effect with OR = 8.52. Of interest is the association of ‘mouth rinse use’ with SP (OR = 5.60). This concurred with findings from a US study which showed association of mouth rinse use with SP [43], though there were many other studies which did not show any evidence of such association [17,18,22,25,26]. This highlights the importance of validating any self-report measures in a given population due to differences in population-specific characteristics, access to dental care, cross-cultural adaptation of questionnaire items and periodontitis prevalence [10,13,15,25]. Among the socio-demographic factors assessed, age, gender and education are significant predictors of SP. Age is considered to be an important risk determinant for periodontitis as it reflects cumulative lifetime disease destruction [44]. Highly educated individuals may be more dentally aware and cultivate positive attitudes regarding self-care and oral hygiene [44].

Disease prediction cannot be accurately achieved from individual screening questions and the performance of a self-reported periodontal questionnaire improved when combined with socio-demographic and risk factors in the model [10], as seen in our study. Our nomogram, which includes both periodontal screening questions and socio-demographic factors, showed excellent discriminatory ability to predict SP with a reported AUC of 0.83. This value is comparable to published range values of 0.64 to 0.85 [23,24], supporting its use for screening purpose. As our nomogram included only six questions, administering the questionnaire with fewer questions would potentially increase recruitment and compliance rates. A nomogram is a user-friendly graphical interface of a multivariable prediction model that can be easily used by clinicians to perform disease prediction at the individual level [31]. The predicted probability for a given individual is calculated based on the summation of scores given for each factor included in the nomogram. Based on the threshold of predicted probabilities set, it can help clinicians quickly identify those at low or high risk of developing SP and refer those with high risk for further clinical assessment and treatment. Different thresholds derived from different combinations of sensitivity and specificity values can be set to classify individuals as low/high risk [45]. In our nomogram, the threshold of 0.59 was set based on the ROC curve analysis with the best balance between sensitivity (70%) and specificity (78%).

To assess the clinical utility of the nomogram, DCA was applied to determine whether the nomogram was useful in identifying individuals who would benefit from a referral to see the periodontist for clinical assessment. This would help in developing a personalized SP risk assessment tool aimed at identifying an individual with a high risk of developing SP. In our study, although a single threshold of predicted probabilities is chosen to simplify the clinical use of the nomogram, improved net benefits across a wide range of threshold probabilities by DCA supports the clinical utility of this nomogram [45]. At the cut-off risk threshold of 0.59, the net benefit of our nomogram is 28%, i.e., the nomogram can identify 28 ‘true positives’ for SP for every 100 individuals in the target population. The use of the nomogram to generate individualized predictions so as to design a personalized treatment plan is widely used in oncology [31]. In dentistry, recent studies have reported the use of the nomogram to screen for severe caries in children [46], vulnerable periodontal condition before orthodontic treatment in patients with Skeletal Class III malocclusion [47], molar survival in patients with periodontitis [48], risk of periimplantitis in treated SP patients [49] and in predicting tooth loss due to periodontal reason [50]. Our study is the first to assess the utility of a nomogram developed from a self-reported periodontal screening instrument in predicting SP. Adoption of the nomogram in SP screening will allow for quick identification of individuals at high risk for developing SP, facilitate communication between patient and healthcare provider and promote early referral for clinical assessment. This method is particularly useful for population mass screening, especially in Singapore, which has a high proportion of individuals with periodontal disease [33].

The strength of our study is that this is the first validity assessment of a self-reported periodontal screening instrument using the nomogram conducted in a sample of a multi-ethnic Southeast Asian population, assessed using a gold standard, full mouth periodontal examination protocol in six sites per tooth using the latest case definition (WWC). In addition, the DCA supports the use of the tool in identifying individuals with high risk of SP. Most of the published validation studies were conducted mainly in the Western population [14,15,17,20,21,22,23] with a few exceptions conducted in the East Asian population [27,28]. None has been conducted in a population such as ours, with a multi-ethnic mix of Chinese, Malays and Indians. Our findings showed that the periodontal screening instrument used in Western countries such as Australia and USA, may be relevant for use as an adjunct to public health surveillance in our local Asian population. Its clinical utility as a personalized SP risk assessment tool is supported by the DCA results. Adopting the nomogram in clinical care will aid the clinicians in identifying those at low and high risk of developing SP. Early detection and management will minimize disease complications.

One limitation of the study is the use of self-reported periodontal questions taken from two different questionnaires, the CDC-AAP and the NSAOH questionnaires. However, these questionnaires have been validated in their respective country of study and we used the NSAOH version of the two questions relating to ‘mouth-rinse use in the last seven days’ and ‘interdental aids use in the last seven days’, as they were phrased such that they were simpler to understand. Another limitation of the study is that only bootstrap internal validation was carried out. As validity of a prediction model may be dependent on the specific study variables it was built from, external validation should be carried out to determine the generalizability of our prediction model [51] in the general population. Our study had included only those who are comfortable in speaking English. Although we believe the results may be extrapolated to the general population as English is one of the four official languages and language policies in Singapore operate based on English as the country’s lingua franca, we plan to do external validation of our nomogram using the self-reporting periodontal screening instrument in the other three official languages (Mandarin, Malay and Tamil). The goal is to develop a simple yet robust instrument to identify individuals with high risk for SP.

## 5. Conclusions

The nomogram based on a self-reported periodontal screening instrument combining six specific predictors demonstrated excellent predictive capability in identifying individuals at risk of developing SP in a multi-ethnic sample of English-speaking Singaporean adults. External validation in the other official languages should be carried to determine its potential use for SP surveillance in the general population.

## Figures and Tables

**Table 1:** Components of the self-reported periodontal screening questionnaire.

Participant’s Demographic and Risk Factor Status	Description
D1. Age	What is your gender?
D2. Gender	Could you tell me your age and date of birth please?
D3. Ethnicity	What is your current ethnicity according to your National Registration Identity Card?
D4. Education	What is your highest education level?
D5. Housing type	What type of house do you live in?
D6. Monthly housing income	Over the last 12 months, what has been the total earnings or income (S$) of the household per month?
D7. Smoking status	Have you been told by a doctor that you have diabetes?
D8. Diabetes status	Which of the following best describes your smoking status (includes cigarettes, cigars and pipes)?
**Periodontal self-report measures**	
Q1. Upper tooth count	There are 16 teeth, including wisdom teeth, in the upper jaw. How many teeth do you have remaining in your UPPER jaw? (implants are regarded as missing)
Q2. Lower tooth count	There are also 16 teeth, including wisdom teeth, in the lower jaw. How many teeth do you have remaining in your LOWER jaw? (implants are regarded as missing)
Q3. Have gum disease	Do you think you have gum disease?
Q4. Lost bone	Has a dental professional ever told you that you have lost bone around your teeth?
Q5. Gum treatment	Have you ever had scaling, root planning, surgery or other treatment for gum disease
Q6. Loose tooth	Have you ever had any teeth that have become loose by themselves without some injury (not baby teeth)?
Q7. Tooth doesn’t look right	During the past three months have you noticed that you have a tooth that doesn’t look right
Q8. Gum health	How would you rate the health of your gums?
Q9. Mouthrinse use	How often in the last 7 days did you use mouthwash or any dental rinse product
Q10. Interdental aids	How often during the last 7 days, did you use dental floss, tape, or an interdental brush to clean between your teeth, other than just to remove food particles stuck between your teeth

**Table 2:** Sample characteristics.

		Age < 45	45 ≤ Age ≤ 54	55 ≤ Age ≤ 64	Age ≥ 65	All
No of Participants	Unit	30	37	49	39	155
**Age**	Mean (SD)	37.77 (5.1)	50.14 (3.2)	59.92 (2.8)	69.72 (4.4)	55.76 (11.8)
**Gender**						
Female	n (%)	14 (46.7%)	17 (45.9%)	24 (49.0%)	17 (43.6%)	72 (46.5%)
Male	n (%)	16 (53.3%)	20 (54.1%)	25 (51.0%)	22 (56.4%)	83 (53.5%)
**Ethnicity**						
Chinese	n (%)	23 (76.7%)	23 (62.2%)	37 (75.5%)	37 (94.9%)	120 (77.4%)
Indian	n (%)	6 (20.0%)	9 (24.3%)	7 (14.3%)	0 (0.0%)	22 (14.2%)
Malay	n (%)	1 (3.3%)	3 (8.1%)	3 (6.1%)	1 (2.6%)	8 (5.2%)
Others	n (%)	0 (0.0%)	2 (5.4%)	2 (4.1%)	1 (2.6%)	5 (3.2%)
**Education**						
Non-tertiary	N (%)	18 (60.0%)	26 (70.3%)	32 (65.3%)	34 (87.2%)	110 (71.0%)
Tertiary	n (%)	12 (40.0%)	11 (29.7%)	17 (34.7%)	5 (12.8%)	45 (29.0%)
**Housing type**						
Public housing	n (%)	26 (86.7%)	28 (75.7%)	32 (69.6%)	24 (63.2%)	110 (72.8%)
Private housing	n (%)	4 (13.3%)	9 (24.3%)	14 (30.4%)	14 (36.8%)	41 (27.2%)
**Monthly household income**						
<SGD $2000	Mean (SD)	4 (18.2%)	3 (9.7%)	10 (29.4%)	11 (55.0%)	28 (26.2%)
SGD $2000–9999	Mean (SD)	13 (59.1%)	24 (77.4%)	18 (52.9%)	8 (40.0%)	63 (58.9%)
≥SGD $10,000	Mean (SD)	5 (22.7%)	4 (12.9%)	6 (17.6%)	1 (5.0%)	16 (15.0%)
**Smoking status**						
Never	n (%)	21 (70.0%)	29 (78.4%)	44 (89.8%)	30 (76.9%)	124 (80.0%)
Current	n (%)	6 (20.0%)	4 (10.8%)	1 (2.0%)	0 (0.0%)	11 (7.1%)
Past	n (%)	3 (10.0%)	4 (10.8%)	4 (8.2%)	9 (23.1%)	20 (12.9%)
**Diabetes status**						
No	n (%)	26 (89.7%)	32 (88.9%)	38 (77.6%)	25 (67.6%)	121 (80.1%)
Yes	n (%)	3 (10.3%)	4 (11.1%)	11 (22.4%)	12 (32.4%)	30 (19.9%)
**Tooth count**	Mean (SD)	26.9 (1.56)	25.32 (3.05)	23.92 (4.42)	18.38 (6.29)	23.44 (5.31)
**WWC classification**						
Healthy	n (%)	8 (26.7%)	2 (5.4%)	11 (22.4%)	1 (2.6%)	22 (14.2%)
Stage I	n (%)	6 (20.0%)	7 (18.9%)	2 (4.1%)	2 (5.1%)	17 (11.0%)
Stage II	n (%)	3 (10.0%)	8 (21.6%)	11 (22.4%)	4 (10.3%)	26 (16.8%)
Stage III	n (%)	12 (40.0%)	15 (40.5%)	22 (44.9%)	19 (48.7%)	68 (43.9%)
Stage IV	n (%)	1 (3.3%)	5 (13.5%)	3 (6.1%)	13 (33.3%)	22 (14.2%)

SD = Standard deviation; WWC: World Workshop on the Classification of Periodontal and Peri-Implant Diseases and Conditions.

**Table 3:** Association of self-reported questions on severe periodontitis according to WWC classifications by logistic regression.

			Univariable		Multivariable	
	Non-Severe Periodontal Disease	Severe Periodontal Disease	OR (95% CI)	*p*-Value	OR (95% CI)	*p*-Value
**Participant’s characteristics**						
**Age**	65	90	1.04 (1.01, 1.07)	0.004	1.06 (1.02, 1.11)	0.003
**Gender**						
Female	40	32	Reference		Reference	
Male	25	58	2.90 (1.50, 5.61)	0.002	2.84 (1.26, 6.64)	0.013
**Ethnicity**						
Chinese	54	66	Reference			
Non-Chinese	11	24	1.79 (0.80, 3.97)	0.155		
**Housing type**						
Public	50	60	Reference			
Private	15	26	1.44 (0.69, 3.02)	0.329		
**Education**						
Non-Tertiary	41	69	Reference		Reference	
Tertiary	24	21	0.52 (0.26, 1.05)	0.068	0.32 (0.12, 0.81)	0.020
**Smoking status**						
Never	52	57	Reference			
Current	3	8	2.43 (0.61, 9.66)	0.206		
Past	10	25	2.28 (1.00, 5.20)	0.050		
**Diabetes status**						
No	56	61	Reference			
Yes	9	28	2.86 (1.24, 6.58)	0.014		
**Self-reported periodontal measures**						
**Upper tooth count (Q1)**	65	90	0.97 (0.89, 1.05)	0.439		
**Lower tooth count (Q2)**	65	90	0.94 (0.85, 1.04)	0.243		
**Gum disease (Q3)**						
No	47	41	Reference			
Yes	13	37	3.26 (1.53, 6.96)	0.002		
**Lost bone (Q4)**						
No	57	63	Reference		Reference	
Yes	4	21	4.75 (1.54, 14.67)	0.007	8.52 (2.33, 41.37)	0.003
**Gum treatment (Q5)**						
No	61	78	Reference			
Yes	2	11	4.30 (0.92, 20.13)	0.064		
**Loose teeth (Q6)**						
No	55	57	Reference		Reference	
Yes	8	32	3.86 (1.64, 9.11)	0.002	4.43 (1.65, 13.22)	0.005
**Tooth doesn’t look right (Q7)**						
No	49	52	Reference			
Yes	15	33	2.07 (1.00, 4.28)	0.049		
**Gum health (Q8)**						
Poor/Fair	21	49	Reference			
Good/Very Good/Excellent	42	39	0.40 (0.20, 0.78)	0.007		
**Mouthrinse use (Q9)**						
No	48	46	Reference		Reference	
Yes	17	44	2.7 (1.35, 5.39)	0.005	5.60 (2.25, 15.53)	<0.001
**Interdental aids (Q10)**						
No	43	63	Reference			
Yes	22	27	0.84 (0.42, 1.66)	0.611		

WWC: World Workshop on the Classification of Periodontal and Peri-Implant Diseases and Conditions.

## Data Availability

The data presented in this study are available from the corresponding upon reasonable request.
